# Retrospective cohort study for thrombocytopenia during concurrent chemoradiotherapy for rectal cancer

**DOI:** 10.3389/fonc.2023.1289824

**Published:** 2024-01-02

**Authors:** Yue Teng, Dapeng Ma, Yan Yan, Jianhao Geng, Zhiyan Liu, Xianggao Zhu, Shuai Li, Yangzi Zhang, Hongzhi Wang, Yong Cai, Haizhen Yue, Yongheng Li, Weihu Wang

**Affiliations:** ^1^ Key Laboratory of Carcinogenesis and Translational Research (Ministry of Education/Beijing), Department of Radiation Oncology, Peking University Cancer Hospital and Institute, Beijing, China; ^2^ Key Laboratory of Carcinogenesis and Translational Research (Ministry of Education), Endoscopy Center, Peking University Cancer Hospital and Institute, Beijing, China; ^3^ State Key Laboratory of Holistic Integrative Management of Gastrointestinal Cancers, Beijing Key Laboratory of Carcinogenesis and Translational Research, Department of Radiation Oncology, Peking University Cancer Hospital and Institute, Beijing, China

**Keywords:** rectal cancer, thrombocytopenia, concurrent chemoradiotherapy, risk factors, clinical predictors, radiation dosimetric parameters, bone marrow

## Abstract

**Background:**

The aim of this article was to establish the clinical prognostic models and identify the predictive radiation dosimetric parameters for thrombocytopenia during concurrent chemoradiotherapy for rectal cancer.

**Methods:**

In this retrospective cohort study, patients with rectal adenocarcinoma undergoing concurrent long-term chemoradiotherapy were included. The primary outcome of interest was grade 2 or higher (2+) thrombocytopenia (platelet(PLT) count <75,000/μL). Secondary outcomes included: grade 1 or higher thrombocytopenia (PLT count<100,000/μL) and the PLT count during chemoradiotherapy and its nadir. The risk prediction model was developed by logistic regression to identify clinical predictors of 2+ thrombocytopenia. Univariate linear regression models were used to test correlations between radiation dosimetric parameters and the absolute PLT count at nadirs.

**Results:**

This retrospective cohort comprised 238 patients. Fifty-four (22.6%) patients developed thrombocytopenia during concurrent chemoradiotherapy, while 15 (6.3%) patients developed 2+ thrombocytopenia. Four independently associated risk factors, including age, Alb level, PLT count, and chemotherapy regimen, were included in the final model and used to form a 2+ thrombocytopenia probability estimation nomogram. The C‐index was 0.87 (95% CI: 0.78–0.96). The calibration plot showed a moderate agreement, and the Brier score was 0.047 (95% CI: 0.025–0.070). The total absolute volume of bone marrow irradiated by 5 Gy, 10 Gy and 15 Gy of radiation (BM-V_5ab_, BM-V_10ab_, BM-V_15ab_), calculated by the volume of bone marrow multiplied by the corresponding Vx, were identified as new predictors. The nadir of PLT was found to be negatively correlated with BM-V_5ab_ (β = -0.062, P =0.030), BM-V_10ab_ (β = -0.065, P =0.030) and BM-V_15ab_ (β = -0.064, P =0.042).

**Conclusion:**

The occurrence of 2+ thrombocytopenia during concurrent chemoradiotherapy for rectal cancer can be predicted by the patient’s baseline status and chemoradiotherapy regimen, and low dose irradiation of bone marrow can affect the level of platelets during the treatment.

## Introduction

Concurrent chemoradiotherapy (CRT) is the standard of care for locally advanced and resectable metastatic rectal cancer. Both radiotherapy and chemotherapy are myelosuppressive. It is known that the pelvic bones, sacrum and lumbar vertebrae, which are located in the irradiated region in rectal cancer, play an important role in hematopoiesis (1). Previous studies have demonstrated that the dose-volume parameters of the pelvic bone marrow (BM) may predict the hematologic toxicity of rectal cancer (2–5). When chemotherapy is prescribed concurrently, the risk of acute hematologic toxicity (HT) increases due to additional BM injury.

Different types of HT pose different risks to the patients, and have different effects on treatment. Among them, cancer therapy-induced thrombocytopenia (CTIT) represents a troublesome toxicity. Platelets are a scarce resource, and recovery of platelets by TPO or IL-11 takes a long time (6, 7). Therefore, once thrombocytopenia occurs, it may cause chemotherapy and radiotherapy dose reduction or treatment delays that are more severe than those related to other types of HT. Treatment breaks may ultimately result in inferior oncological outcomes. In addition, CRT is not the end of rectal cancer treatment. Neoadjuvant chemoradiotherapy has lasting effects on the bone marrow, which are demonstrable during adjuvant chemotherapy, and patients experience more severe thrombocytopenia in adjuvant chemotherapy (8, 9). In addition, under total neoadjuvant therapy (TNT) treatment strategy, thrombocytopenia occurring during concurrent chemoradiotherapy may result in delayed or missed chemotherapy cycles and treatment breaks in consolidation chemotherapy, which follows closely. The incidence rate of thrombocytopenia is also greater in consolidation chemotherapy than in CRT (10).

We assumed that a better understanding of thrombocytopenia would be conducive to its prevention and management. The aim of this article was to analyze the incidence, pattern and risk factors (including clinical factors, radiation dose and target volumes) for thrombocytopenia during CRT in patients with rectal cancer, based on retrospective data from our institution.

## Methods

### Patient population

For this retrospective cohort study, consecutive patients undergoing concurrent long-term chemoradiotherapy with a capecitabine and oxaliplatin (XELOX) regimen for histologically confirmed rectal adenocarcinoma at Beijing Cancer Hospital between Apr 1, 2015, and Nov 30, 2021, were included. Other inclusion criteria were as follows: tumor located <12 cm from the anal verge; T2-4 N0-2, M0 or resectable M1 according to the 8^th^ edition of the American Joint Committee on Cancer TNM Classification; and age ≥ 18 and ≤ 75 years. The exclusion criteria were as follows: diagnosis of double primary cancer, history of any other tumor, previous radiation, or low baseline platelet(PLT) count (<100,000/μL).

### Radiotherapy planning and chemotherapy regimen

Patients received radiotherapy planning computed tomography (CT) and pelvic magnetic resonance (MR) scanning with 5 mm slice thickness. The MR images were subsequently fused with the CT images. Patients were treated in the prone position with a full bladder and an empty rectum Simultaneous integrated boost intensity-modulated radiation therapy (SIB-IMRT) was performed. The gross tumor volume (GTV) was defined as the primary tumor and metastatic lymph nodes. The clinical target volume (CTV) included: mesorectal and presacral regions, internal iliac and obturator lymph node drainage areas, ≥2 cm margins from the cephalic and caudal extents of the primary lesion in the rectum and 1-2 cm margins around all identified areas of invasion. The IMRT regimens included: a total dose of 50.6 Gy (GTV)/41.8 Gy (CTV) in 22 fractions, or a total dose of 50 Gy (GTV)/45 Gy (CTV) in 25 fractions. This schedule was described in our previous work (11–14).

The concurrent chemotherapy regimen was daily capecitabine (1650 mg/m2/day, orally twice daily during the RT course) and oxaliplatin. The dosage of oxaliplatin (50 mg/m2/qw, 85 mg/m2/q2w, or 100-130 mg/m2/q3w) was prescribed.

### Bone marrow delineation and dose calculation

**Figure 1:**
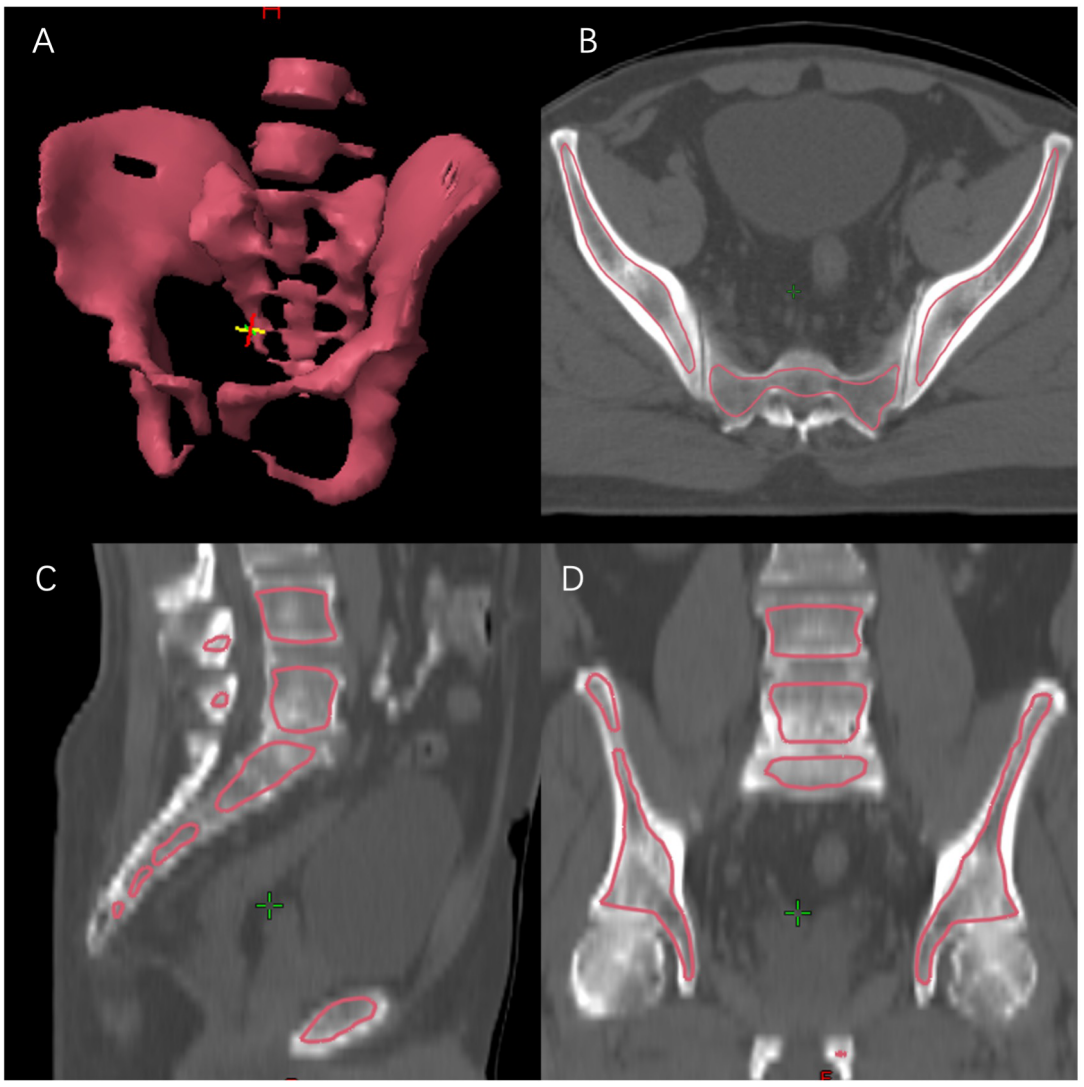
Bone marrow delineation. **(A)** 3D reconstruction; **(B)** transverse section; **(C)** sagittal section; **(D)** coronal section.

To quantify the volume of pelvic bone marrow irradiated in the IMRT plans, the medullary space within the pelvic bones and lumbar spines was contoured. BM delineation was performed at the eclipse planning station. The window was adjusted to the bone range to contour the low-density region inside the bone. The pelvic BM was delineated as the inner cavity of bone from the top of the L4 vertebra to the bottom of the ischial tuberosity, using previously described methods (**Figure 1**). The total volume of PTV (V_PTV_), total volume of BM (V_BM_), mean dose of BM (BM-D_mean_), and volume of BM receiving different radiation dose from 5 Gy to 40 Gy (BM-V_5_ to BM-V_40_) were estimated (recorded as percentages). Additionally, the total absolute volume of bone marrow irradiated by different doses was defined as BM-V_5ab_ to BM-V_40ab_ (recorded as absolute volume), calculated by: V_BM_ multiplied by BM-V_5_ to BM-V_40_.

### Study endpoints and safety assessment

The primary outcome of interest was the grade 2 or higher (2+) thrombocytopenia (PLT<75,000/μL) during chemoradiotherapy and within 14 days of the end of the last treatment, according to the Common Terminology Criteria for Adverse Events (CTCAE) Version 5.0. Secondary outcomes included: grade 1 of higher thrombocytopenia (PLT<100,000/μL), PLT count during radiotherapy and its nadir, effects of thrombocytopenia on chemotherapy dose and dose delays, and radiotherapy delays and reduction. Patients underwent complete blood counts at baseline and weekly during chemoradiotherapy. Liver and renal functions were assessed at least every 2 weeks. Hematologic toxicity was graded according to the National Cancer Institute Common Terminology Criteria for Adverse Events.

### Statistical analyses

Multivariate logistic regression analysis was performed to identify predictors of 2+ thrombocytopenia. All eligible variables with univariable P values <0.1 were incorporated into a multivariate regression model and sequentially removed using backward elimination techniques.

The risk prediction model of thrombocytopenia was developed by logistic regression. A final model selection was performed by a backward stepwise selection process with the Akaike information criterion (AIC). The model was presented as a nomogram. We assessed nomogram model performance by examining overall accuracy (Brier score), calibration (calibration plots and Hosmer–Lemeshow calibration test), and discrimination (Harrell C index and its 95% CI).

Correlations between radiation dosimetric parameters and absolute PLT counts at the nadirs was tested by univariate linear regression models. P<0.05 was defined as statistically significant. Statistical analyses were performed with SPSS Statistics software (version 24, IBM Corp., Armonk, NY) and R software (version 4.2.2).

## Results

### Clinical characteristics

This retrospective cohort comprised 238 patients. **Table 1** shows the baseline patient and clinical characteristics. The median age of the cohort was 57 (range, 30-73) years. Two hundred and twenty-nine (96.6%) patients had stage T3-4 disease, and 230 (96.6%) patients had node-positive disease. Eighteen (7.6%) patients received induction chemotherapy. All patients received concurrent chemotherapy with different regimens, and the dosing frequency of oxaliplatin was decided by the physician. Most patients (71.4%) received intravenous oxaliplatin every two weeks.

**Table 1:** Patient demographics.

Characteristics N=238	N (%)
Sex	
Male	183 (76.9)
Female	55 (23.1)
Age, years	
Median age (range)	57 (30-73)
T Stage	
cT2	9 (3.8)
cT3	146 (61.3)
cT4	83 (34.9)
N Stage	
cN0	8 (3.4)
cN+	230 (96.6)
Resectable metastasis	
No	181 (76.1)
Yes	57 (23.9)
Lung	12
Liver	27
Non-reginal lymph nodes	19
Induction chemotherapy	
Yes	18 (7.6)
No	220 (92.4)
Concurrent chemotherapy regimen *	
Qw	8 (3.4)
Q2w	170 (71.4)
Q3w	60 (25.2)
Further treatment	
Surgery	187 (78.6)
Chemotherapy	25 (10.5)
Watch and wait	16 (6.7)
Unknown	10 (4.2)

*Intravenous oxaliplatin chemotherapy regimen: Qw, 50 mg/m2 applied every week; Q2w, 85 mg/m2 applied every two weeks; Q3w, 100-130 mg/m2 applied every three weeks.

### Incidence of thrombocytopenia and dynamic changes in PLT count during CRT

Fifty-four (22.7%) patients developed thrombocytopenia during CRT, while 15 (6.3%) patients developed grade 2+ thrombocytopenia. Among the patients who developed thrombocytopenia (N=54), treatment was affected by the disease in 17 (31.5%) patients, including radiotherapy (N=5) or chemotherapy interruption (N=10), chemotherapy delay (N=6), and radiotherapy dose reduction (N=1).

**Figure 2:**
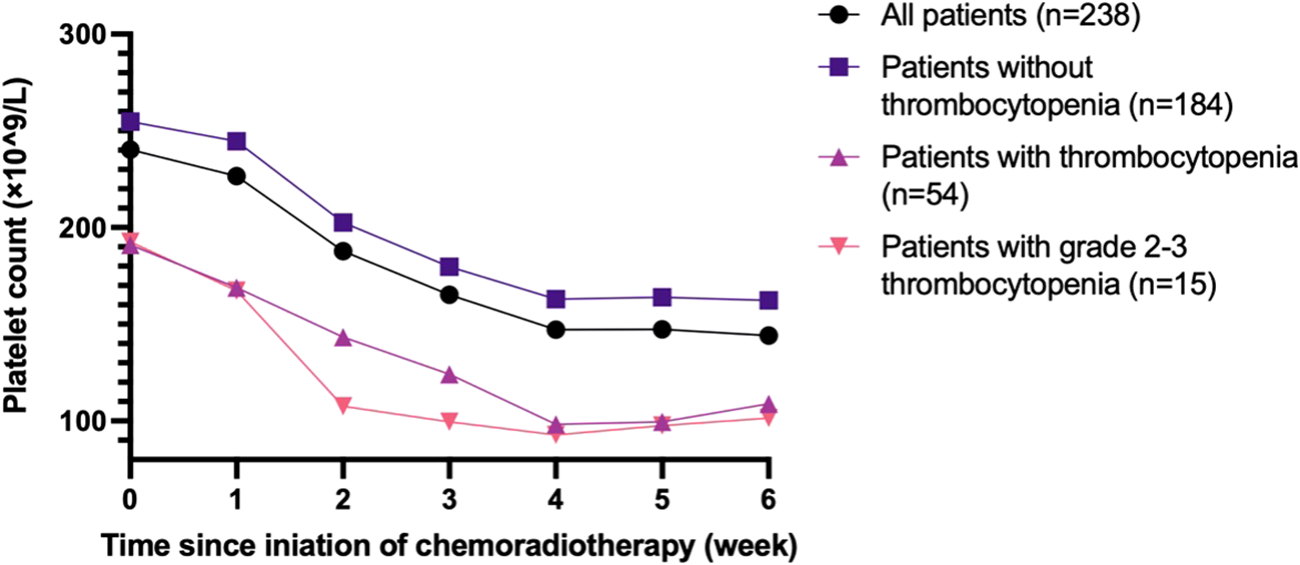
Platelet count during concurrent chemoradiotherapy.

**Figure 2** demonstrates the changes in the PLT count during chemoradiotherapy in every week. Generally, the PLT count decreased gradually as the treatment progressed, declining rapidly between week 1 and week 4. Patients who developed thrombocytopenia during treatment typically had a lower PLT count at baseline.

### Risk factors associated with grade 2+thrombocytopenia

On univariate analysis, older age, low albumin (Alb) level at baseline, low PLT count at baseline, induction chemotherapy, and intravenous oxaliplatin chemotherapy every three weeks were statistically significantly associated with increased risks of grade 2+ thrombocytopenia (**Table 2**). On multivariate analysis, older age, low PLT count at baseline, and intravenous oxaliplatin chemotherapy every three weeks remained as significant, independent predictors of the risk of disease (**Table 2**). In the risk receiver-operating characteristic (ROC) curve analysis of the PLT count for grade 2+ thrombocytopenia, the area under the ROC curve (AUC) indicated the prognostic value of the PLT count, with an AUC of 0.718 (P = 0.005), and a cutoff value of 194.5×10^9^/L.

**Table 2:** Analysis of factors associated with grade 2-3 thrombocytopenia.

Predictor of 2+ thrombocytopenia	Univariate analysis		Multivariate analysis	
	OR (95% CI)	P value	OR (95% CI)	P value
Sex (male)	0.22 (0.03-1.74)	0.152		
Age (per year)	1.09 (1.02-1.17)	0.016	1.12 (1.02-1.22)	0.018
Body mass index	1.01 (0.86-1.19)	0.931		
NRS 2002 ≥2	2.40 (0.80-7.25)	0.119		
Alb	0.85 (0.75-0.97)	0.017	0.84 (0.69-1.02)	0.071
PLT	0.98 (0.97-0.99)	0.004	0.98 (0.97-0.99)	0.012
Induction chemotherapy	5.43 (1.53-19.25)	0.009	0.38 (0.07-1.96)	0.247
Chemotherapy regimen *	4.82 (1.64-14.20)	0.004	8.19 (2.31-29.00)	0.001
V_PTV_	1.00 (1.00-1.00)	0.772		
V_BM_	1.00 (1.00-1.01)	0.329		
BM-V_5_	1.05 (0.9 4-1.17)	0.399		
BM-V_10_	1.02 (0.95-1.10)	0.562		
BM-V_5ab_ #	1.01 (1.00-1.01)	0.169		
BM-V_10ab_ #	1.00 (1.00-1.01)	0.173		
BM-D_mean_	1.000 (0.998-1.001)	0.702		

Alb, albumin level at baseline; PLT, platelet count at baseline; BM, bone marrow; V_PTV:_, PTV volume; V_BM_, bone marrow volume; NA, not applicable.

*Frequency of intravenous oxaliplatin chemotherapy: Q3w vs. Q2w.

#Total absolute volume of bone marrow irradiated by ≥5 Gy or 10 Gy, calculated as the volume of bone marrow multiplied by BM-V5 or BM-V10.

### Prediction model development and validation

**Figure 3:**
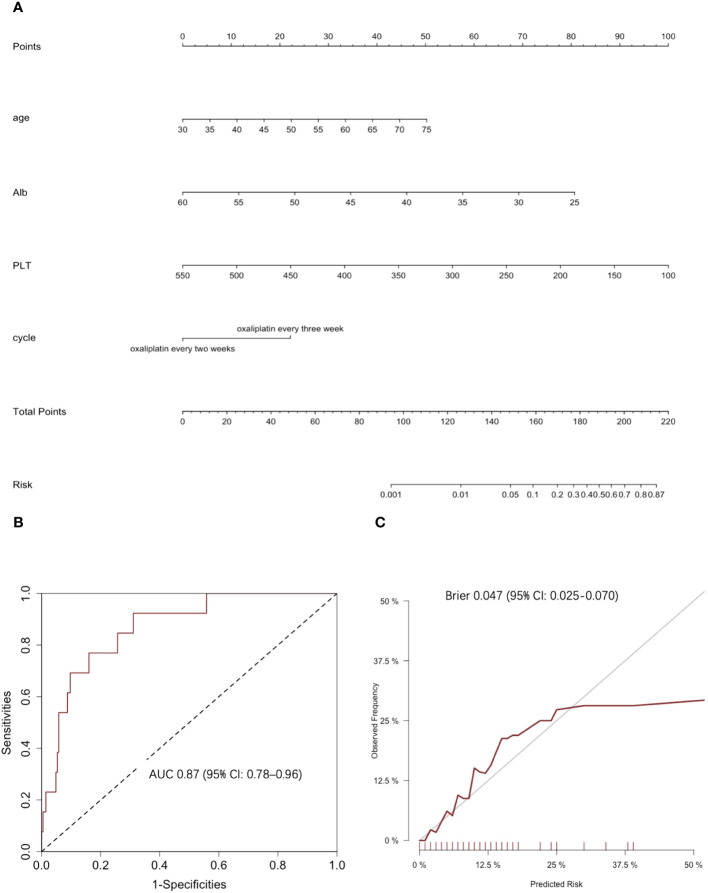
Clinical prediction model of 2+ thrombocytopenia. **(A)** The nomogram for predicting 2+ thrombocytopenia: This nomogram provides a method for calculating the risk of developing 2+ thrombocytopenia. To use, locate the patient’s age, draw a line straight up to the points axis to establish the score associated with that age. Repeat for the other three covariates (Alb level at baseline, PLT count at baseline and chemotherapy cycle). Add the score of each covariate together and locate the total score on the total points axis. Draw a line straight down to the risk axis to obtain the probability. **(B)** ROC curves and corresponding AUC statistics. **(C)** Calibration plots: nomogram-predicted 2+ thrombocytopenia is plotted on the x-axis, with observed 2+ thrombocytopenia on the y-axis. Dashed lines along the diagonal line through the origin point represent the perfect calibration models in which the predicted probabilities are identical to the observed probabilities.

Based on the results of the backward stepwise selection process with the AIC, four risk factors, including age, Alb level, PLT count, and chemotherapy regimen, were included in the final model and used to form a 2+ thrombocytopenia probability estimation nomogram (**Figure 3A**).

The predictive accuracy for 2+ thrombocytopenia as measured by the C‐index was 0.87 (95% CI: 0.78–0.96) (**Figure 3B**). The Hosmer‐Lemeshow calibration test was significant (χ2 = 2.27, p = 0.97) and the calibration plot for the probability of 2+ thrombocytopenia showed a moderate agreement between the actual observed outcome and the prediction by the nomogram (**Figure 3C**). The overall prediction performance was good, with a mean Brier score of 0.047 (95% CI: 0.025–0.070).

### PLT count nadir and its associations with radiation dosimetric parameters

**Table 3** further illustrates the role of radiation dosimetric parameters in predicting the PLT count nadirs during CRT. Traditional radiation dosimetric parameters, including V_5_-V_40_, BM-D_Mean_, Volume of PTV and BM volume, were not associated with a lower PLT count nadir. However, the total absolute volume of bone marrow irradiated by 5 Gy, 10 Gy and 15 Gy (BM-V_5ab_, BM-V_10ab_, BM-V_15ab_), calculated as the volume of bone marrow multiplied by the corresponding V_x_, were predictors of the PLT count nadir. The nadir of PLT was found to be negatively correlated with BM-V_5ab_ (β = -0.062, P =0.030), BM-V_10ab_ (β = -0.065, P =0.030) and BM-V_15ab_ (β = -0.064, P =0.042).

**Table 3:** Linear regression model parameters associated with PLT count nadirs.

Factors	PLT count nadir	
	β	P value
BM-V_5_	-0.824	0.057
BM-V_10_	-0.525	0.146
BM-V_15_	-0.281	0.347
BM-V_20_	-0.143	0.626
BM-V_25_	-0.037	0.905
BM-V_30_	0.108	0.759
BM-V_35_	0.281	0.509
BM-V_40_	0.419	0.454
V_PTV_	0.003	0.791
V_BM_	-0.037	0.237
BM-D_mean_	-0.004	0.661
BM-V_5ab_	-0.062	0.030 *
BM-V_10ab_	-0.065	0.030 *
BM-V_15ab_	-0.064	0.042 *
BM-V_20ab_	-0.064	0.078
BM-V_25ab_	-0.064	0.146
BM-V_30ab_	-0.058	0.306
BM-V_35ab_	-0.038	0.618
BM-V_40ab_	-0.010	0.930

BM, bone marrow; V_PTV_, volume of PTV; V_BM_, volume of bone marrow;

BM-V_xab_: Total absolute volume of bone marrow irradiated by ≥x Gy, calculated as the volume of bone marrow multiplied by BM-V_x_. *p<0.05.

## Discussion

In the present study, we focused on thrombocytopenia induced by CRT of rectal cancer, investigating its clinical characteristics of occurrence, impact on treatment and relevant risk factors.

Chemotherapy induced thrombocytopenia (CIT) is a common hematologic toxicity in long-term chemotherapy that has been noted by medical oncologists. The incidence of CIT observed in colorectal cancer patients treated with the adjuvant XELOX regimen was 72.3% (15), and the incidence of grade 3-4 thrombocytopenia was 5.3%-18.5% (16–18) in previous studies. However, the narrow definition of CIT can no longer cover the current diversified tumor treatment methods. In the guidelines of the Chinese Society of Clinical Oncology (CSCO) 2022, the concept of CTIT was proposed. CTIT, cancer therapy-induced thrombocytopenia, is an extensional concept of CIT, which was extended from chemotherapy to the all kinds of antitumor treatment, including radiotherapy, targeted therapy and immunotherapy.

CTIT during radiotherapy for rectal cancer has not received enough attention. This is because traditional neoadjuvant long-term CRT has a short treatment cycle and long interval before surgery for patient recovery. However, under the trend of the total neoadjuvant treatment (TNT) strategy (19–21), preoperative chemoradiation is actually more intensive, which would theoretically lead to more severe thrombocytopenia. The associated dual-therapy modality results in a greater number of hematologic toxicities than monotherapy in the neoadjuvant treatment of rectal cancer (22). Therefore, this enhanced combination treatment strategy has increased radiotherapists’ interest in thrombocytopenia. In our study, 54 (22.6%) patients developed grade 1-3 thrombocytopenia during CRT. This is lower than the data reported for the TNT strategy (10). In our cohort, 49 (20.5%) patients received TNT-like CRT plus induction or consolidation chemotherapy. Among these patients, 17 (34.7%) developed CTIT during TNT-like treatments, and most of these patients (12 patients, 24.5%) developed 2+ thrombocytopenia. These results suggest that greater attention needs to be paid to the clinical impact of thrombocytopenia after adding higher-intensity neoadjuvant chemotherapy.

According to current guidelines, only grade 2+ thrombocytopenia requires clinical intervention. The low incidence of grade 2+ thrombocytopenia may lead to underestimation of impact of thrombocytopenia by radiotherapists. In real-world clinical practice, however, the treatment of patients affected by thrombocytopenia is more than expected. In a large sample size study of a secondary analysis of data from prospective clinical trials, 62% of CIT adverse events (AEs) led to chemotherapy dose delay or change and/or discontinuation in metastatic colorectal cancer patients (23). In our cohort, the treatments of 19 (35.2%) patients were affected by CTIT, and for 5 patients, both chemotherapy and radiotherapy were impacted. Based on our analysis, PLT declined gradually within four weeks after the beginning of CRT and stabilized in the later periods of treatment. Among the 13 patients who experienced chemotherapy interruption, most (84.6%) dropped the last cycle. Thus, patients who received intravenous oxaliplatin chemotherapy every three weeks experienced a greater dose reduction. Sequential chemotherapy or adjuvant chemotherapy could compensate for the dose reduction in CRT, but the efficacy is unknown. Relatively, the radiotherapy regimens were only slightly impacted. One patient had the last 2 fractions dropped, and 2 patients had radiotherapy delays of more than 3 days due to CTIT. However, in many patients in our cohort, the date of radiotherapy delay was not consistent with the date of thrombocytopenia in our cohort. Radiotherapy, unlike chemotherapy, is a continuous process. Patients may not see the doctor in time to suspend radiotherapy when thrombocytopenia occurs, or they may make their own decision to stop radiotherapy because of their own worries. This should convince doctors of the need for more detailed patient education.

Recognizing the risk factors for thrombocytopenia helps in the conduction of reasonable management and prevention. Therefore, we further investigated the predictive factors for grade 2+ thrombocytopenia. Risk factors for CIT found by previous studies included tumor type, stage, chemotherapy regimen, chemotherapy cycles, and high lactate dehydrogenase levels (24–26). In our study, patient-specific factors were also significantly associated with the incidence of 2+ thrombocytopenia, including age and baseline Alb level, which revealed the nutrition level of the patient. Baseline PLT count remained a strong predictor in multivariate analysis, as shown in **Figure 1**. Chemotherapy, as we expected, made patients prone to thrombocytopenia. Specifically, different frequencies of intravenous oxaliplatin chemotherapy led to different susceptibilities to thrombocytopenia for the patients. Patients receiving intravenous oxaliplatin every two weeks had a lower incidence rate of 2+ thrombocytopenia than those receiving oxaliplatin every three weeks. In addition, patients often had their last cycle of chemotherapy dropped once they began experiencing thrombocytopenia, as described earlier. From this point of view, oxaliplatin 85 mg/m2 applied every two weeks may be a safer choice. Induction chemotherapy was associated with 2+ thrombocytopenia in univariate analysis, as we expected, but did not reach statistical significance in multivariate analysis. This may be due to its small sample size (n=18) and its effect on PLT count at baseline (the beginning of CRT).

Our prediction model performs well in predicting a low risk of 2+ thrombocytopenia, which means that these patients may receive CRT or even total neoadjuvant therapy without excessive worries of severe thrombocytopenia. However, this model failed to filter out patients with high risk, so that it is hard to determine who is suitable for prophylactic use of TPO. This may be due to the relatively low risk of thrombocytopenia in CRT of rectal cancer because most patients only received CRT instead of total neoadjuvant therapy during the study period. However, this model may show a greater significance in the era of TNT. Other regression modeling techniques will also be explored to determine whether predictive accuracy can be further improved.

It is generally believed that pelvic bone marrow irradiation will affect hematopoietic function, thus theoretically leading to thrombocytopenia. In many studies involving pelvic radiotherapy, thrombocytopenia was mixed with leukopenia and/or anemia for analysis or was neglected directly in risk factor analysis (4, 5, 27, 28). However, treatments are different between thrombocytopenia and leukopenia, and thrombocytopenia usually requires a longer recovery process than leukopenia and thus needs to be independently predicted. The conclusions among previous studies have been inconsistent. In a study of cervical cancer patients treated with CRT, no radiation dosimetric parameters were recognized as risk factors for thrombocytopenia or PLT count nadirs (29). However, in another rectal cancer cohort, the V_5_ and V_10_ of the BM predicted the PLT count nadir% (specified as a percentage of the baseline value) (3). V5 was also identified as a factor associated with PLT nadir (2). Patients with V_40_>23% (lower pelvic bone marrow) had a higher rate of grade 2+ thrombocytopenia in another study (30). The reasons for these conflicting findings may include the different contouring strategies for bone marrow and the various endpoints defined in different studies. Active BM delineation on MR images seems to be more accurate than delineation on CT (2). The dose-volume parameters under this strategy may serve as better predictors (3), but more evidence is still needed. In this study, we contoured the medullary space for radiation dosimetric analysis (31). Interestingly, the traditional radiation dosimetric parameters did not serve as significant predictors. However, when we multiplied the V_5~15_ and BM volume, the new parameters BM-V_5ab_, BM-V_10ab_ and BM-V_15ab_ were significantly associated with the PLT count nadir and had a lower p value in the univariate analysis in predicting grade 2+ thrombocytopenia. This new parameter is the total absolute volume of bone marrow irradiated by ≥5-15 Gy. This result suggested that the decrease in platelet counts is associated with low-dose irradiation of the bone marrow, but the traditional radiation dosimetric parameters failed to reveal this relationship.

Our study has its limitations. Given that this was a single-center retrospective study and the incidence of 2+ thrombocytopenia was relatively low, the results need to be validated in a larger prospective cohort. In addition, during the study period, most patients received only CRT instead of total neoadjuvant therapy. Thrombocytopenia may be a more important issue in the future, but its importance was not fully reflected in this study. Extra attention should be given to the treatment of relatively high-risk patients identified in our study, especially in total neoadjuvant therapy. Due to the small number of positive cases, this model was not externally validated. Future efforts will seek to test our model performance in external validation using other patient databases.

Nevertheless, this is a comprehensive and detailed analysis of thrombocytopenia induced by both chemotherapy and radiotherapy in rectal cancer patients. We analyzed the incidence rate, pattern and risk factors for thrombocytopenia during CRT to ensure the safe and timely treatment of patients in the future. The occurrence of 2+ thrombocytopenia during concurrent chemoradiotherapy for rectal cancer can be predicted by the patient’s baseline status and chemoradiotherapy regimen, and low dose irradiation of bone marrow can affect the level of platelets during the treatment.

## Data availability statement

The original contributions presented in the study are included in the article/supplementary material. Further inquiries can be directed to the corresponding authors.

## Ethics statement

Ethics approval was not required due to the retrospective nature of the study.

## Author contributions

YT: Data curation, Formal Analysis, Methodology, Writing – original draft. DM: Data curation, Writing – original draft. YY: Formal Analysis, Writing – original draft. JG: Writing – review & editing. ZL: Resources, Writing – review & editing. XZ: Resources, Writing – review & editing. SL: Methodology, Writing – review & editing. YZ: Resources, Software, Writing – review & editing. HW: Writing – review & editing. YC: Writing – review & editing. HY: Formal analysis, Writing – review & editing. YL: Conceptualization, Funding acquisition, Investigation, Writing – review & editing. WW: Conceptualization, Supervision, Writing – review & editing.
